# Comprehensive Scanning of Prophages in *Lactobacillus*: Distribution, Diversity, Antibiotic Resistance Genes, and Linkages with CRISPR-Cas Systems

**DOI:** 10.1128/mSystems.01211-20

**Published:** 2021-06-01

**Authors:** Zhangming Pei, Faizan Ahmed Sadiq, Xiao Han, Jianxin Zhao, Hao Zhang, R. Paul Ross, Wenwei Lu, Wei Chen

**Affiliations:** a State Key Laboratory of Food Science and Technology, Jiangnan University, Wuxi, Jiangsu, China; b School of Food Science and Technology, Jiangnan University, Wuxi, Jiangsu, China; c National Engineering Research Center for Functional Food, Jiangnan University, Wuxi, Jiangsu, China; d International Joint Research Center for Probiotics & Gut Health, Jiangnan University, Wuxi, Jiangsu, China; e APC Microbiome Ireland, University College Cork, Cork, Ireland; Pacific Northwest National Laboratory; Canadian Food Inspection Agency

**Keywords:** CRISPR-Cas system, *Lactobacillus* prophage, antibiotic resistance genes, genomic diversity

## Abstract

Prophage integration, release, and dissemination exert various effects on host bacteria. In the genus *Lactobacillus*, they may cause bacteriophage contamination during fermentation and even regulate bacterial populations in the gut. However, little is known about their distribution, genetic architecture, and relationships with their hosts. Here, we conducted prophage prediction analysis on 1,472 genomes from 16 different *Lactobacillus* species and found prophage fragments in almost all lactobacilli (99.8%), with 1,459 predicted intact prophages identified in 64.1% of the strains. We present an uneven prophage distribution among *Lactobacillus* species; multihabitat species retained more prophages in their genomes than restricted-habitat species. Characterization of the genome features, average nucleotide identity, and landscape visualization presented a high genome diversity of *Lactobacillus* prophages. We detected antibiotic resistance genes in more than 10% of *Lactobacillus* prophages and validated that the occurrence of resistance genes conferred by prophage integration was possibly associated with phenotypic resistance in Lactobacillus plantarum. Furthermore, our broad and comprehensive examination of the distribution of CRISPR-Cas systems across the genomes predicted type I and type III systems as potential antagonistic elements of *Lactobacillus* prophage.

**IMPORTANCE** Lactobacilli are inherent microorganisms in the human gut and are widely used in the food processing industries due to their probiotic properties. Prophages were reportedly hidden in numerous *Lactobacillus* genomes and can potentially contaminate entire batches of fermentation or modulate the intestinal microecology once they are released. Therefore, a comprehensive scanning of prophages in *Lactobacillus* is essential for the safety evaluation and application development of probiotic candidates. We show that prophages are widely distributed among lactobacilli; however, intact prophages are more common in multihabitat species and display wide variations in genome feature, integration site, and genomic organization. Our data of the prophage-mediated antibiotic resistance genes (ARGs) and the resistance phenotype of lactobacilli provide evidence for deciphering the putative role of prophages as vectors of the ARGs. Furthermore, understanding the association between prophages and CRISPR-Cas systems is crucial to appreciate the coevolution of phages and *Lactobacillus.*

## INTRODUCTION

Temperate phages follow a lysogenic cycle after infecting bacterial cells and integrate their genomes into the host bacterial chromosome without causing disruption. These latent phages are known as prophages, which usually remain in a quiescent state, and their genomes are replicated with the host genome. However, in some cases, prophages can be induced into a lytic cycle by stressful environmental factors (1), and as a result, their DNA is excised from bacterial genomes (2), replicated, and packaged into complete phage particles, facilitating horizontal gene transfer (HGT) (3). Prophages are involved in several bacterial life processes; the expression of prophage functional genes can confer survival advantages on lysogens in adverse environments (4, 5); virulent genes carried by prophages not only increase the virulence of the host bacteria (6) but can even convert a nonvirulent strain into a pathogenic strain (7); antibiotic resistance genes (ARGs) can be disseminated via phage-mediated transduction (8), possibly enhancing bacterial pathogenicity. Therefore, it is becoming increasingly evident that elucidation of the roles of prophages in the bacterial life cycle is pertinent to the complete understanding of bacterial physiology, evolution, and population dynamics.

Prophages and temperate phages have been much less studied than virulent phages because of the uncertainty in prophage induction (9). Although mitomycin C (MMC) treatment is considered the most effective induction method for prophages, the sensitivity of different bacteria to different levels of MMC differs greatly (10); thus, prophage isolation and identification are not sufficiently efficient (11). However, in recent years, with the rapid development of next-generation sequencing, prophage prediction based on the genome has become a research hot spot. Many studies aimed to reveal the prevalence and diversity of prophages across different bacterial genera/species. Two recent studies in large cohorts analyzed more than 1,000 bacterial genome sequences of Salmonella (12) and Streptococcus (13), respectively, and reported the prevalence of prophage regions in strains belonging to both species. Some other studies also showed a high occurrence of prophages in Clostridioides difficile (14), Helicobacter pylori (15), and Klebsiella pneumoniae (16).

Prophages are also widely distributed among probiotic strains commonly used in dairy fermentation, such as *Lactococcus* (17), *Bifidobacterium* (18), and *Lactobacillus*. Ventura et al. (19) reported at least four prophage-like entities in the genome of a single strain of Lactobacillus plantarum. In a study by Brandt et al. (20), all 11 randomly selected Lactobacillus rhamnosus strains carried the phage Lc-Nu genome. The occurrence of prophage-related sequences has been investigated within Lactobacillus brevis (21), Lactobacillus ruminis (22), and Lactobacillus gasseri (23), but the degree of prevalence varies among the three species. It has been proved that prophage release is one of the main sources of virulent phage infection in dairy starter cultures (24), which may cause slow and failed fermentation. On the other hand, in the gut, Lactobacillus reuteri (a model gut symbiont) prophage was found to be induced by a fructose-enriched diet (25). Therefore, the understanding of lysogeny in *Lactobacillus* is essential to comprehend the consequences of prophage-induced bacterial cell lysis in the fermentation industry and the influence on the community structure and function of the intestinal commensals. However, due to the limited availability of assembled *Lactobacillus* genome sequences, genus-wide analyses of the genomic diversity and population structure of *Lactobacillus* prophages have not yet been reported.

This study included a total of 1,472 strains belonging to 16 *Lactobacillus* species; we reported the discovery of over 4,000 prophage fragments and 1,459 intact prophage regions, which provided comprehensive insights into the distribution of *Lactobacillus* prophages. All intact prophages were clustered based on average nucleotide identity (ANI) analysis of their genomes to assess the population structure and their genetic diversity and complexity. By annotating *Lactobacillus* prophage genomes, we described the distribution of predicted ARGs located in prophage regions and proposed potential risks of ARG-carrying prophages in *L. plantarum*. Finally, we performed a comprehensive prediction of the clustered regularly interspaced short palindromic repeats (CRISPR) and associated genes (Cas) among all 1,472 *Lactobacillus* genomes, in an attempt to explore the associations between CRISPR-Cas systems and prophages in *Lactobacillus*.

## RESULTS

### Identification within genomes of 1,472 *Lactobacillus* strains presents a broad and uneven prophage distribution.

**FIG 1:**
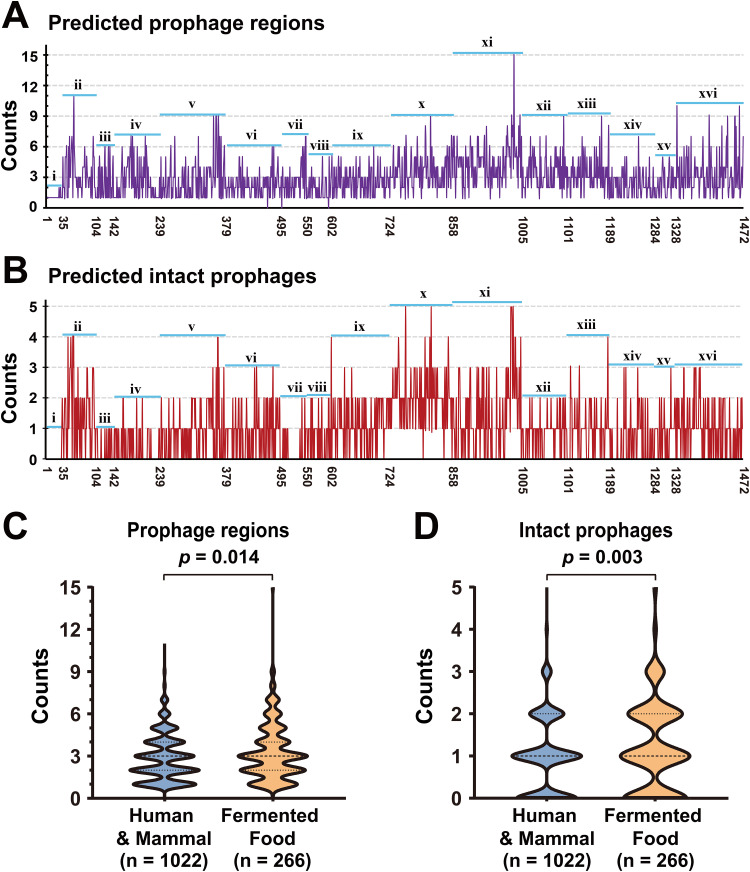
The distribution of prophages in 1,472 *Lactobacillus* genomes. (A) The counts of all predicted prophage regions in each *Lactobacillus* genome. (B) The counts of predicted intact prophages in each *Lactobacillus* genome. The *x* axis presents strains arranged from left to right according to the order in Table S1A. In panels A and B, labels marked i to xvi represent strains belonging to 16 species of *Lactobacillus*, i, L. acidophilus; ii, L. brevis; iii, *L. bulgaricus*; iv, L. crispatus; v, *L. fermentum*; vi, *L. (para)gasseri*; vii, L. helveticus; viii, L. johnsonii; ix, *L. mucosae*; x, *L. plantarum*; xi, *L. (para)casei*; xii, L. rhamnosus; xiii, L. reuteri; xiv, *L. ruminis*; xv, *L. sakei*; and xvi, *L. salivarius*. The number of predicted intact prophages among the 16 groups was compared using the nonparametric Kruskal-Wallis test for multiple independent samples, and pairwise adjustment significance two-tailed *P* values are visualized in Fig. S1. (C) Comparison of the number of predicted prophage regions between the human/mammal group and the fermented food group. (D) Comparison of the number of predicted intact prophages between the human/mammal group and the fermented food group. Statistical significance tests were performed using the nonparametric Mann-Whitney U test, and the two-tailed *P* values were calculated.

To determine a comprehensive prophage distribution in the genus *Lactobacillus*, the genomes of 1,472 selected strains from 16 species were screened for prophage identification using PHASTER (prediction results are detailed in Table S2). Overall, 4,360 prophage regions were identified in 99.8% (1,469/1,472) of the genome sequences being analyzed. Only three strains did not carry any prophage fragments, indicating that prophages are highly prevalent in different *Lactobacillus* species. The number of prophages varied greatly among the strains, with 0 to 15 prophage regions found in each strain (Fig. 1A). The highest number of prophages (15) occurred in Lactobacillus paracasei strain EG9, isolated from cheese. Among the 4,360 predicted prophage regions, most of them were marked as questionable or incomplete prophage fragments, and 1,459 prophage regions were marked as intact prophages and were distributed in 64.1% (944/1,472) of the *Lactobacillus* strains, ranging from 1 to 5 per strain (Fig. 1B). Six strains [four *L.* (*para*)*casei* strains and two *L. plantarum* strains] carried the highest number (5) of intact prophages. The presence of a high number of intact prophages shows that potential functional prophages (complete, inducible, or transferable) are widely distributed in the genus *Lactobacillus*, and these predicted intact prophages were used as main subjects for further analysis.

10.1128/mSystems.01211-20.6 TABLE S2 The prophage prediction results of 1,472 *Lactobacillus* genomes. Download Table S2, XLSX file, 0.3 MB. Copyright © 2021 Pei et al. 2021 Pei et al. https://creativecommons.org/licenses/by/4.0/ This content is distributed under the terms of the Creative Commons Attribution 4.0 International license.

10.1128/mSystems.01211-20.1 FIG S1 The pairwise adjustment significance two-tailed *P* values of prophage distribution between *Lactobacillus* species. Download FIG S1, TIF file, 1.1 MB. Copyright © 2021 Pei et al. 2021 Pei et al. https://creativecommons.org/licenses/by/4.0/ This content is distributed under the terms of the Creative Commons Attribution 4.0 International license.

10.1128/mSystems.01211-20.5 TABLE S1 (A) Specific information of 1,472 *Lactobacillus* strains/genomes involved in this study. (B) Information of 81 *Lactobacillus* phages downloaded from NCBI. Download Table S1, XLSX file, 0.1 MB. Copyright © 2021 Pei et al. 2021 Pei et al. https://creativecommons.org/licenses/by/4.0/ This content is distributed under the terms of the Creative Commons Attribution 4.0 International license.

The prophage distribution for each *Lactobacillus* species is extremely uneven, as summarized in Table 1; L. brevis, *L. plantarum*, and *L.* (*para*)*casei* strains harbored a significantly higher number of intact prophages (Fig. S1; *P < *0.05) with higher occurrence rates than most of the other species. These three species are generally present in a wide range of habitats, including human feces, fermented vegetables, dairy products, and liquor. In contrast, Lactobacillus acidophilus, Lactobacillus bulgaricus, Lactobacillus crispatus, Lactobacillus helveticus, and *L. ruminis*, which only occupy a restricted habitat, carry few intact prophages with generally lower occurrence rates. Additionally, let us consider *L.* (*para*)*casei* and L. rhamnosus as examples; they are two species in a close genetic relationship (26), and have similar occurrence rates of intact prophage; however, *L.* (*para*)*casei* occupies various habitats and carried a higher number of prophages than L. rhamnosus (Table 1). These results suggest that, in the genus *Lactobacillus*, species that tended to occupy multiple habitats retain more intact prophages in their genomes than other species.

**TABLE 1:** The number of prophages within each *Lactobacillus* species

Species (no. of strains)	Common habitats	No. of prophage fragments			No. of intact prophages			Occurrence of intact prophages (%)
		Min	Max	Mean	Min	Max	Mean	
L. acidophilus (*n* = 35)	Human gut	1	2	1.06	0	1	0.03	2.9
L. brevis (*n* = 69)	Multiple	1	11	3.45	0	4	1.54	78.2
L. bulgaricus (*n* = 38)	Dairy products	1	6	2.64	0	1	0.41	32.0
L. crispatus (*n* = 97)	Human vagina	1	7	2.21	0	2	0.18	31.6
L. fermentum (*n* = 140)	Human gut/fermented food	1	9	2.72	0	4	0.91	63.8
L. (para)gasseri (*n* = 116)	Human gut	0	6	2.24	0	3	0.91	67.2
L. helveticus (*n* = 54)	Dairy products	0	7	2.65	0	2	0.20	16.7
L. johnsonii (*n* = 53)	Human gut	0	5	1.83	0	2	0.70	56.6
L. mucosae (*n* = 122)	Human gut	1	6	2.55	0	4	1.16	79.5
L. plantarum (*n* = 134)	Multiple	1	9	3.54	0	5	2.03	98.5
L. (para)casei (*n* = 147)	Multiple	1	15	4.39	0	5	1.39	72.1
L. rhamnosus (*n* = 96)	Human gut	1	9	3.41	0	2	0.85	74.0
L. reuteri (*n* = 88)	Human gut	1	9	3.35	0	4	0.99	72.7
L. ruminis (*n* = 95)	Human gut	1	7	2.28	0	3	0.59	43.2
L. sakei (*n* = 44)	Fermented food	1	5	2.43	0	3	0.77	65.9
L. salivarius (*n* = 144)	Human gut/saliva/blood	1	10	3.38	0	3	1.03	69.4
Total (*n* = 1,472)		0	15	2.97	0	5	0.99	64.1

To further investigate whether the number of intact prophages carried by *Lactobacillus* strains is related to their habitat, 1,288 *Lactobacillus* strains with definite isolation sources were divided into the “human/mammal” group (*n *= 1,022) and “fermented food” group (*n *= 266), and the differences in the number of prophages between the two groups were compared. The results demonstrated that the number of prophages harbored by *Lactobacillus* strains belonging to the fermented food group was significantly higher than that harbored by strains belonging to the human/mammal group; this was observed for all predicted prophage regions (Fig. 1C;* P* = 0.014) and intact prophages (Fig. 1D;* P* = 0.003).

### General genome features and integration sites of *Lactobacillus* prophages.

**FIG 2:**
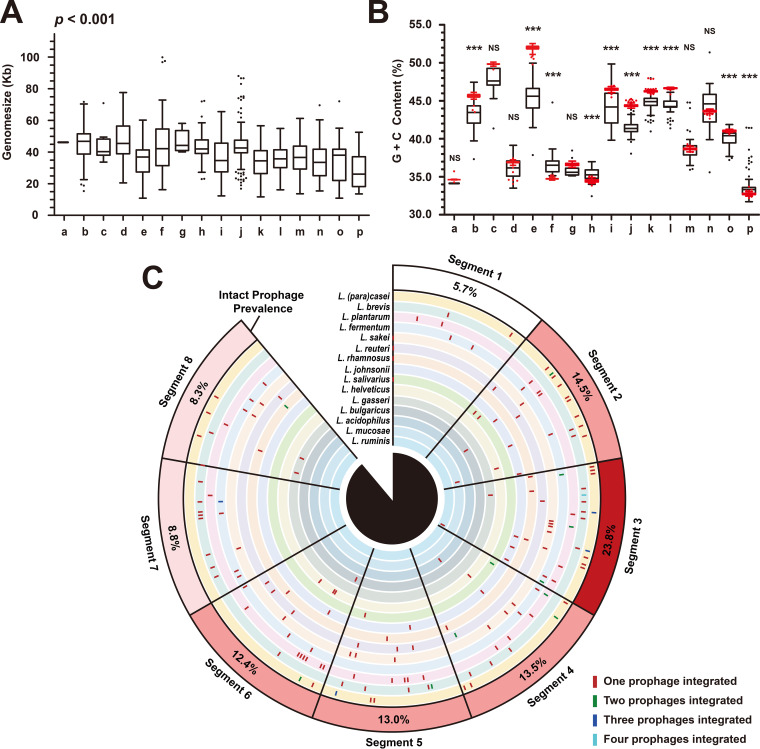
General genome features and integration sites of *Lactobacillus* prophages. (A) Genome size of prophages. (B) GC content of prophages (black box plot) and their hosts (red box plot). The groups labeled a to p represent prophages belonging to 16 species of *Lactobacillus* genus, a, L. acidophilus; b, L. brevis; c, *L. bulgaricus*; d, L. crispatus; e, *L. fermentum*; f, *L. (para) gasseri*; g, L. helveticus; h, L. johnsonii; i, *L. mucosae*; j, *L. (para)casei*; k, *L. plantarum*; l, L. reuteri; m, L. rhamnosus; n, *L. ruminis*; o, *L. sakei*; p, *L. salivarius*. Each group of data in panels (A) and (B) used Tukey’s HSD test for plotting the whiskers and outliers. The genome sizes of prophages in 16 groups were compared using the nonparametric test for multiple independent samples—the Kruskal-Wallis test. The GC content of prophages and host bacteria in each group were compared using two independent samples *t* test (*n* < 30) or *z* test (*n* ≥ 30), as appropriate. Two-tailed *P* values were calculated, NS, *P > *0.05; *, 0.01 < *P ≤ *0.05; ***, *P ≤ *0.001. (C) Prophage integration sites within *Lactobacillus* genomes. At least one complete genome was available from 15 *Lactobacillus* genomes. The names of the bacterial genomes used for analysis are provided in Data Set S1, Tab1, and the data of integration sites are provided in Table S2.

In total, we revealed 1,459 putatively intact *Lactobacillus* prophages in this data set; their average genome size was 38.31 ± 14.50 kb (median ± interquartile range), ranging from 10.57 kb (*L. fermentum* prophage FTDC8312P8) to 99.82 kb (L. gasseri prophage MV_22P3). Significant differences in genome size were observed between groups of prophages belonging to different host species (Fig. 2A;* P* = 0.001). Seven out of sixteen groups showed outliers, especially for *L.* (*para*)*casei* prophages (group j), with a total of 29 outliers (13 longer genomes and 16 shorter genomes), revealing a high level of variation in genome sizes among prophages within and between bacterial species. Further, guanine and cytosine content (GC content) is an important feature of genomic nucleotide content, which can be used as an indicator to assess the evolution of microorganisms (27). It varies considerably in *Lactobacillus* prophages, from 29.5% to 51.3%. When prophages were grouped according to their host species, their GC contents were different than the mean values of host bacteria GC contents (Fig. 2B). We observed significant differences between phage and host GC content in 11 out of the 16 groups. For *Lactobacillus* species with higher GC contents (groups b, e, i, j, k, l, and o), the GC contents of their prophages were significantly lower than the GC contents of their host, whereas prophages of some bacterial species with lower GC contents (groups a, f, h, and p) had significantly higher GC contents than their host. In addition, all 1,459 putative intact prophage genomes were compared with publicly available *Lactobacillus* phage sequences in GenBank, and only 16.2% of them (236/1,459) matched with 29 published genomes (Fig. S2), indicating that most of the intact prophages predicted in this study were probably new. Remarkably, even though the hosts of most prophages were identical to those of the matched *Lactobacillus* phages, we still found prophages from different *Lactobacillus* species whose sequences aligned with the same public phage sequence, suggesting that cross-species transmission of prophages is possible in the genus *Lactobacillus*.

10.1128/mSystems.01211-20.2 FIG S2 Comparisons between 1,459 putative intact prophages and publicly available *Lactobacillus* phage sequences in GenBank. Download FIG S2, TIF file, 1.5 MB. Copyright © 2021 Pei et al. 2021 Pei et al. https://creativecommons.org/licenses/by/4.0/ This content is distributed under the terms of the Creative Commons Attribution 4.0 International license.

**FIG 3:**
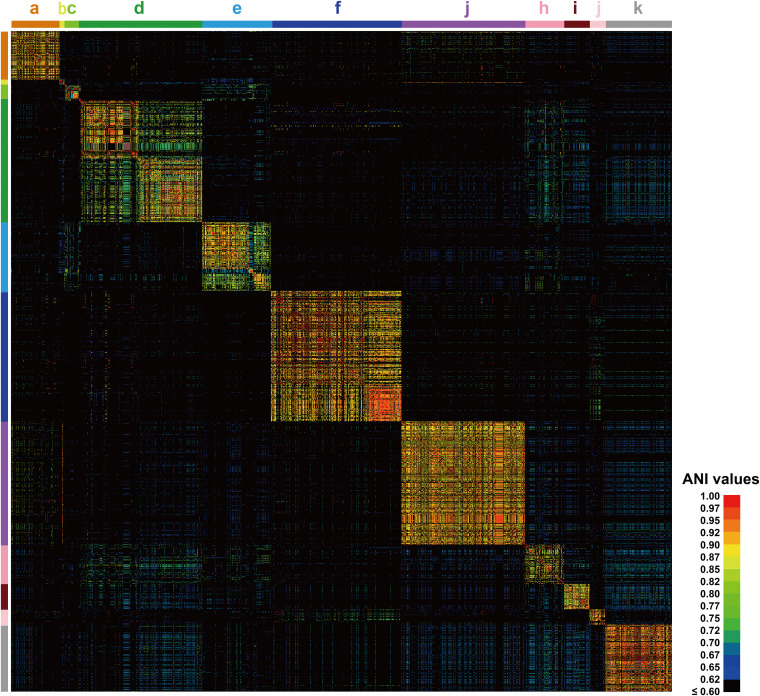
Pairwise ANI values across 1,459 *Lactobacillus* prophage genomes. Prophages are arranged from top to bottom and left to right in their order in Data Set S1, Tab2. The clusters labeled a to k’ represent prophages carried by 15 different host species of *Lactobacillus*, a, L. brevis; b, *L. bulgaricus*; c, L. crispatus; d, *L. (para)gasseri*, L. helveticus, and L. johnsonii; e, *L. fermentum* and *L. mucosae*; f, *L. (para)casei* and L. rhamnosus; g, *L. plantarum*; h, L. reuteri; i, *L. ruminis*; j, *L. sakei*; k, *L. salivarius*. The colors in the heat map represent values with a gradient from blue (low identity) to red (high identity).

**FIG 4:**
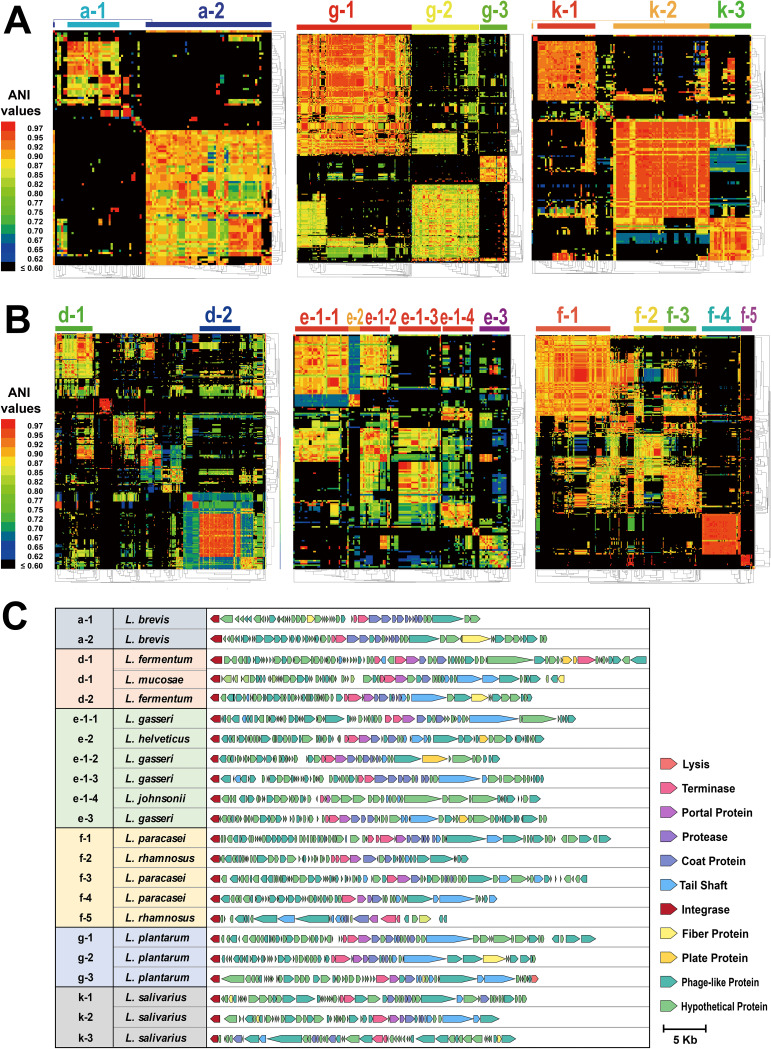
*Lactobacillus* prophages vary greatly. (A) Clustering of pairwise ANI values across prophages of six clusters. The average-linkage hierarchical cluster method based on Pearson distance was used to cluster rows and columns in all clusters. The subclusters are graphed by color bars and labels at the top of heat maps. The colors in the heat map represent values with a gradient from blue (low identity) to red (high identity). The name orders of prophages on the top of each cluster (from left to right) are listed in Data Set S1, Tab2. (B) Genomic organization of representative intact prophages from different subclusters. Each gene is colored based on the known or putative function.

**FIG 5:**
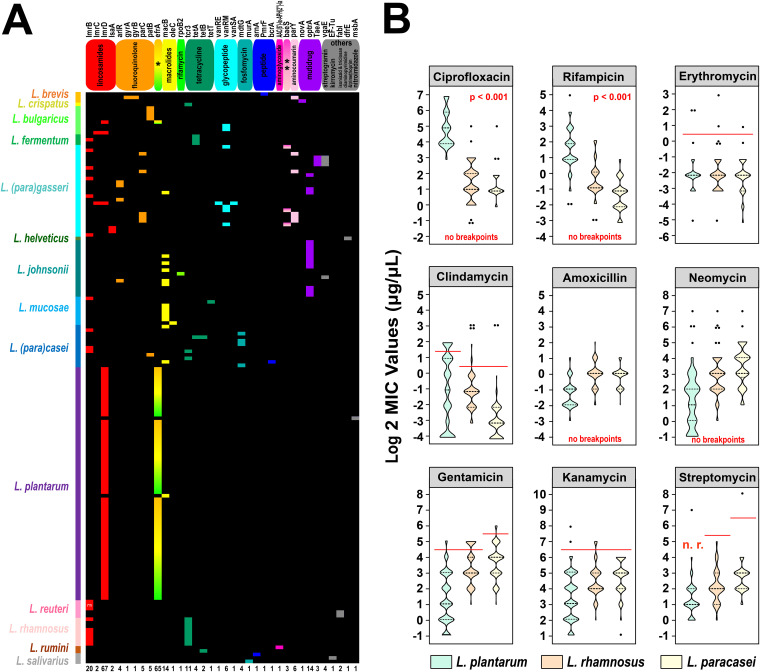
*Lactobacillus* prophages are associated with antibiotic resistance. (A) The occurrence and distribution of ARGs across *Lactobacillus* prophages. Heat map illustrating the distribution of ARGs across 162 *Lactobacillus* prophages. The name order of prophages with ARGs (from top to bottom) is listed in Data Set S1, Tab3. The gene names and corresponding resistant antibiotics (types) are displayed at the top of the heat map, whereas the number of different ARGs is indicated at the bottom. *, *efrA* confers resistance to fluoroquinolone, macrolides, and rifamycin; **, *baeS* confers resistance to aminoglycoside and aminocoumarin. The names and host species (color bar) of the *Lactobacillus* prophages are indicated on the left side, whereas the gene copy numbers are listed on the right side. The code “*2” indicates that L. reuteri prophage FNXL81L1P2 carries two copies of *lmrB*. The ARG prediction results are provided in Data Set S1, Tab4. (B) Distribution of MIC values of 9 antibiotics for 115 *L. plantarum*, 121 *L. paracasei*, and 71 L. rhamnosus strains. The red lines represent microbiological breakpoints recommended by the EFSA. Statistical significance tests were performed using the nonparametric Kruskal-Wallis test.

10.1128/mSystems.01211-20.9 DATA SET S1 (Tab1) The genomes used in integrated site analysis. (Tab2) Ranking of the predicted intact *Lactobacillus* prophages in Fig. 3 and 4A. (Tab3) Ranking of the predicted intact *Lactobacillus* prophages with ARGs in Fig. 5A. (Tab4) The ARG predictions among predicted intact *Lactobacillus* prophages. (Tab5) The result of CRISPR spacer OTU clustering. (Tab6) The result of the local BLAST alignment between CRISPR spacers and *Lactobacillus* (pro)phages. Download Data Set S1, XLSX file, 1.8 MB. Copyright © 2021 Pei et al. 2021 Pei et al. https://creativecommons.org/licenses/by/4.0/ This content is distributed under the terms of the Creative Commons Attribution 4.0 International license.

Investigating the integration sites of prophages may help comprehend the infectiousness and host specificity of *Lactobacillus* phages; hence, we examined the location of prophage integration sites within the genomes of 15 *Lactobacillus* species for which at least one complete genome was available (L. crispatus has no complete genome). The relative integration sites of 193 predicted intact prophages (from 106 complete genomes) are presented in Fig. 2C. In general, the location of intact prophages varied greatly among the different *Lactobacillus* species or different strains of the same species; nonetheless, we discovered that there were more than two phages integrated on the same site. This indicates that most *Lactobacillus* temperate phages may have strict host specificity, but there is also a small number of phages that can infect different strains. Moreover, each complete genome was divided into eight equal-length segments, and the percentages of intact prophages located in each segment were quantified. Interestingly, we observed strong preferences of prophage insertion in segments 2 to 6, especially in segment 3, in which 23.8% of the intact prophages were inserted. In addition, Lactobacillus sakei, L. reuteri, L. rhamnosus, and Lactobacillus salivarius each had a prophage inserted into the replication origin of the bacterial genome.

### Clustering of *Lactobacillus* prophage showed specificity dependent on the host species.

The evolutionary relationship among the prophages was studied through genome sequence homology and genome composition. ANI determines the similarity between two genome sequences at the gene level; we calculated ANI values to estimate the genetic relatedness among these prophages. All 1,459 prophage genomes were aligned in pairs; the results show that only 10.1% (215,014/2,128,681) of the pairs had values higher than 60.0% in 1,459 prophages, suggesting a generally low genomic similarity among *Lactobacillus* prophages. After arranging the matrix data and visualizing the heatmap of pairwise ANI values (Fig. 3), it was found that the 1,459 prophages were located in 11 independent clusters, and the ANI values of prophages existing in different clusters were found to be generally low. Of the 11 clusters, 8 (clusters a, b, c, g, h, i, j, and k) were composed of prophages from a single *Lactobacillus* species. In addition, *L.* (*para*)*gasseri*, L. helveticus, and Lactobacillus johnsonii prophages constituted cluster d; Lactobacillus fermentum and Lactobacillus mucosae prophages formed cluster e; *L.* (*para*)*casei* and L. rhamnosus prophages formed cluster f. Based on this, we deduced that the genome similarity between *Lactobacillus* prophages is closely related to the genetic relationship of their hosts in all probabilities. The farther apart the evolutionary relationship between host species, the lower the similarity between the prophages. In contrast, for those species with a close genetic relationship, such as *L.* (*para*)*casei* and L. rhamnosus; *L. fermentum* and *L. mucosae*; and *L.* (*para*)*gasseri*, L. helveticus, and L. johnsonii, their prophages showed relatively higher pairwise ANI values, further illustrating that cross-species transmission of *Lactobacillus* prophages is common but probably only happens among species with a close genetic relationship. According to the above-described findings, we speculate that the main factor responsible for the genetic diversity of *Lactobacillus* prophages was the host bacterial species. Moreover, owing to the presence of multiple relatively discrete species in the genus *Lactobacillus*, the whole *Lactobacillus* prophage population also reflects a considerable genetic diversity and numerous relatively independent taxa.

### The diversity of *Lactobacillus* prophages in different clusters varies greatly.

Prophages in each cluster shared highly recognizable DNA similarities; however, many prophages within the same cluster showed an array of genetic dissimilarities. To determine the extent to which prophages in a single cluster deserved to be linked or be segregated, we selected 8 independent clusters (clusters a, d, e, f, g, h, i, and k), all containing at least 50 prophages. Each cluster’s pairwise ANI values were calculated and clustered in rows and columns using the average-linkage hierarchical cluster method based on Pearson distance. The heatmaps showed that all 8 test clusters were highly diverse, each containing 2 to 5 subclusters with more than 10 individuals. In Clusters a, g, h, i, and k, prophages were grouped into several relatively independent subclusters, indicating that the prophages harbored by L. brevis, *L. plantarum*, L. reuteri, *L. ruminis*, and *L. salivarius* have distinct evolutionary origins (Fig. 4A; Fig. S3A). In contrast, for prophages in clusters d, e, and f, although several distinct subclusters could also be observed, the remaining prophages were relatively disordered (Fig. 4B). Considering that these three clusters were all composed of prophages from species with a close genetic relationship, there were complicated evolutionary directions of phylogeny in these prophage populations, presumably due to the diversified host strains, making the genetic evolutionary relationship more complex and variable.

10.1128/mSystems.01211-20.3 FIG S3 (A) Clustering of pairwise ANI values across prophages of L. reuteri (cluster h) and *L. ruminis* (cluster i). (B) Genomic organization of representative intact prophages from different subclusters. (C) Genomic organization of each intact prophage from *L. plantarum* KP and *L. paracasei* BL23. Download FIG S3, TIF file, 2.9 MB. Copyright © 2021 Pei et al. 2021 Pei et al. https://creativecommons.org/licenses/by/4.0/ This content is distributed under the terms of the Creative Commons Attribution 4.0 International license.

To visually and intuitively demonstrate the extent of differences in the structures of *Lactobacillus* prophages for each cluster or species, the landscapes of representative intact prophages were depicted, and we selected one for each subcluster of 8 major clusters (clusters a, d, e, f, g, h, i, and k), one for 3 minor clusters (clusters b, c, and j), and the only L. acidophilus prophage, YTP1. As shown in Fig. 4C and Fig. S3B, it was discovered that both *Lactobacillus* intact prophages demonstrated well-conserved patterns in genome composition and organization, and 31 representative prophages possessed genes involved in lysogeny (integrase), DNA replication, DNA packaging, and morphogenesis (portal, capsid, tail, or other structural proteins). However, regardless of the parameter considered (whether genome size, alignment of genes, or the similarity of genes encoding a specific function), the diversity of *Lactobacillus* prophages in different species, clusters, or subclusters varied greatly. Multiple predicted intact prophage regions within the same strain also showed variations in structural composition; for example, 5 intact prophages from *L. paracasei* BL23 and 5 intact prophages from *L. plantarum* KP were located in 4 and 2 different subclusters, respectively. Evidence for genes shared between the same strain was limited (Fig. S3C). Certainly, it is known that there are still many genes labeled as phage-like proteins or hypothetical proteins at present; therefore, the delineations of these *Lactobacillus* prophages remain problematic. Overall, the results of ANI analyses, clustering, and landscape visualization indicated that the *Lactobacillus* prophage population driven by host species has a rich diversity with an abundance of different types of prophages.

### Prophage may be an important vector for ARGs in *Lactobacillus*.

A total of 259 potential ARGs were identified in 11.1% (162/1,459) of *Lactobacillus* prophages, with each prophage carrying 1 to 4 ARGs (Fig. 5A). Species for which a relatively high rate of ARGs was detected in prophages include *L. bulgaricus* (66.7%, 8/12), L. johnsonii (43.2%, 19/44), *L.* (*para*)*gasseri* (24.8%, 26/105), and *L. plantarum* (24.3%, 66/272). These ARGs belonging to 36 different categories included lincosamides (*lmrB*, *lmrC*, *lmrD*, *lsaA*), fluoroquinolone (*arlR*, *gyrA*, *gyrB*, *parC*, *patB*), macrolides (*macB*, *oleC*), rifamycin (*rpoB2*), tetracycline (*tcr3*, *tetA*, *tetB*, *tetT*), glycopeptide (*vanRE*, *vanRM*, *vanSA*), fosfomycin (*mdtG*, *murA*), peptide (*arnA*, *PmrF*, *bcrA*), aminoglycoside [*aac(6’)-le-aph(2’)-la*], aminocoumarin (*parY*), and multidrug (*efrA*, *baeS*, *novA*, *optrA*, *TaeA*). Among all potential ARGs, *efrA* and *lmrD* had the highest occurrence rates, and 64 intact prophages harbored by different *L. plantarum* strains were detected to carry these two genes. More interestingly, the genes *efrA* and *lmrD* were adjacent, and all 134 *L. plantarum* strains carried this one special gene cluster, which was not found in all other *Lactobacillus* species. Besides 64 *L. plantarum* strains that had this gene cluster identified on intact prophages, 19 strains had it on questionable or incomplete prophages, and it was also found in the adjacent segments of the predicted prophage regions of 20 other *L. plantarum* strains (Table S3A). By further comparison with the nonredundant protein sequence database, this gene cluster was identified as ATP-binding cassette transporter; thus, we considered that it is very likely to be an antibiotic efflux pump introduced into *L. plantarum* via integration of prophages.

10.1128/mSystems.01211-20.7 TABLE S3 (A) The distribution of the *efrA*&*lmrD* resistance gene cluster carried by 134 *L. plantarum*. (B) Antibiotic sensitivity (MIC) of three *Lactobacillus* species against nine antibiotics. Download Table S3, XLSX file, 0.1 MB. Copyright © 2021 Pei et al. 2021 Pei et al. https://creativecommons.org/licenses/by/4.0/ This content is distributed under the terms of the Creative Commons Attribution 4.0 International license.

To further explore whether this gene cluster helps *L. plantarum* strains in developing a resistance phenotype to certain antibiotics, we selected 4 antibiotics related to *efrA* and *lmrD* (erythromycin [*efrA*-macrolides], clindamycin [*lmrD*-lincosamides], ciprofloxacin [*efrA*-fluoroquinolone], and rifampin [*efrA*-rifamycin], and 5 other antibiotics commonly used in susceptibility testing [gentamicin, kanamycin, streptomycin, neomycin [aminoglycosides], and amoxicillin [penicillin]). We then measured the MIC values of these 9 antibiotics for *L.* (*para*)*casei*, L. rhamnosus (as a control species whose strains lacked this unique gene cluster), and *L. plantarum*. As shown in Fig. 5B and Table S3B, for five antibiotics with definite microbiological breakpoints, most of the tested strains in all three species were susceptible to them, and no significant differences were observed among the three species. It is suggested that this potential antibiotic efflux pump is not involved in resistance to clindamycin, erythromycin, and aminoglycosides. The microbiological breakpoints of the other four antibiotics for lactobacilli have not yet been determined. For neomycin, another aminoglycoside antibiotic, the MIC values for most strains of the three *Lactobacillus* species were less than 32 μg/ml. Referring to the breakpoints of the other three aminoglycoside antibiotics, we speculated that strains of these three species were also sensitive to neomycin. For amoxicillin, based on the ranges of MIC values for the three species, we found that the majority of strains could be considered susceptible to amoxicillin to the same extent, so we assumed that the sensitivity of *L. plantarum* to neomycin and amoxicillin is also not related to this gene cluster. Interestingly, however, for the last two antibiotics—ciprofloxacin and rifampin, both implicated in the *efrA* gene—all *L. plantarum* strains showed an extremely high tolerance, while the MIC values for the other two species strains were significantly lower than those for *L. plantarum* (Fig. 5B; *P* = 0.01). Therefor, we suggest that this putative antibiotic efflux pump can probably confer ciprofloxacin and rifampin resistance on the host.

In addition, we also checked all genomes of strains that showed resistance to any of the antibiotics, intending to verify the degree of agreement between the genotypes of prophages and phenotypes of strains. Among the 52 resistant *Lactobacillus* strains (5 to erythromycin, 26 to clindamycin, 25 to gentamicin, 7 to kanamycin, and 1 to streptomycin; 12 strains were resistant to two or more antibiotics), 13 strains harbored intact prophages with potential ARGs. The *lmrD* gene implicated in resistance to lincosamides was detected in prophages of 9 *L. plantarum* strains (FCQNA38L1, DHLJZD24L1, FZJTZ31M7, VCQKX1M2, VCQYC1M1, VCQZX1M2, VHuNHHMY9L1, VJXSRYG1L1, and VJXSRYG2L1), which were phenotypically resistant to clindamycin. These concordances between resistance phenotypes and genotypes also demonstrate that prophages may be an important vector for ARGs in *Lactobacillus* and participate in developing substantial resistance phenotypes to several antibiotics.

### Type I/III CRISPR-Cas systems may be the antagonistic element of *Lactobacillus* prophage.

**FIG 6:**
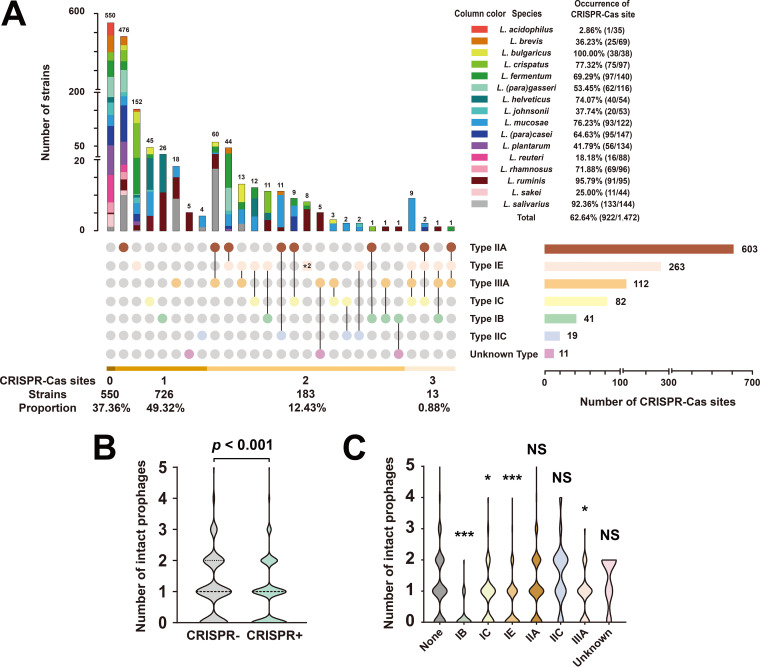
Association between CRISPR-Cas systems and the number of intact prophages in *Lactobacillus*. (A) The occurrence and distribution of the CRISPR-Cas system in 1,472 *Lactobacillus* genomes. The upset plot illustrates the occurrence of CRISPR-Cas sites across 16 species of *Lactobacillus*, the distribution of the CRISPR-Cas system in *Lactobacillus*, and the abundance of each subtype of the CRISPR-Cas system. The code “*2”represents the strains carrying two complete type IE CRISPR-Cas systems. (B) Comparison of the number of intact prophages in *Lactobacillus* genomes with or without a CRISPR-Cas system. (C) Comparisons of the number of intact prophages in *Lactobacillus* genomes with each subtype and without a CRISPR-Cas system. Statistical significance tests were performed using the nonparametric Mann-Whitney U test, and the two-tailed *P* values were calculated; NS, *P > *0.05; *, 0.01 < *P ≤ *0.05; ***, *P ≤ *0.001.

Bacteria have developed multiple and sophisticated antiphage defense systems that can activate cleavage and incorporation of the exogenous genes into the bacterial genome (28). The CRISPR-Cas system is the most common antiphage defense strategy, which confers adaptive immunity to host bacteria through memorization and recognition of past phage attacks (29). Numerous putative CRISPR arrays were predicted in *Lactobacillus* genomes, and further investigation of Cas genes revealed a total of 1,131 complete CRISPR-Cas loci in 62.6% (922/1,472) of the genomes tested (Fig. 6A and Table S4). A huge difference in the detection rate of the CRISPR-Cas system for 16 different species of *Lactobacillus* was found, ranging from 2.9% to 100.0%, and interestingly, species with low detection rates of the CRISPR-Cas system, such as L. brevis (36.3%) and *L. plantarum* (41.8%), had the highest occurrence rates of intact prophages among all *Lactobacillus* species. Conversely, most of the species with high detection rates of the CRISPR-Cas system, such as *L. bulgaricus* (100.0%), *L. ruminis* (95.8%), L. crispatus (77.3%), and L. helveticus (74.0%), had the lowest occurrence rates of intact prophages, except L. acidophilus. Meanwhile, from the perspective of all *Lactobacillus* species, we found that CRISPR-positive strains carried significantly fewer intact prophages than did CRISPR-negative strains (Fig. 6B). These results suggest that CRISPR-Cas systems play a vital role in the inhibition of prophage integration into lactobacilli.

10.1128/mSystems.01211-20.8 TABLE S4 The prediction results of CRISPR-Cas systems among 1,472 *Lactobacillus* genomes. Download Table S4, XLSX file, 0.1 MB. Copyright © 2021 Pei et al. 2021 Pei et al. https://creativecommons.org/licenses/by/4.0/ This content is distributed under the terms of the Creative Commons Attribution 4.0 International license.

Three major CRISPR-Cas system types (types I, II, and III) were discovered in *Lactobacillus*, including six definite subtypes (IB, IC, IE, IIA, IIC, IIIA) and one uncertain subtype, revealing the type diversity of the CRISPR-Cas system in *Lactobacillus*. The type IIA system, the most widely distributed in *Lactobacillus*, was detected in 41.0% (603/1,472) of *Lactobacillus* genomes. The occurrence rates of type I and type III systems were 23.3% and 7.6%, respectively (Fig. 6A). Although type IIA CRISPR-Cas systems are relatively widespread across the genus *Lactobacillus*, there was no difference in the number of intact prophages between type IIA CRISPR-positive and -negative strains (Fig. 6C). It appeared that *Lactobacillus* strains harboring the type IIA CRISPR-Cas defense are not efficiently resistant toward temperate phage infections and integration in *Lactobacillus*. Instead, type I/III CRISPR-positive strains both were found to harbor significantly fewer prophages than CRISPR-negative strains, suggesting that type I/III CRISPR-Cas systems may be the antagonistic elements against *Lactobacillus* prophage.

### Association between CRISPR spacers and prophages.

**FIG 7:**
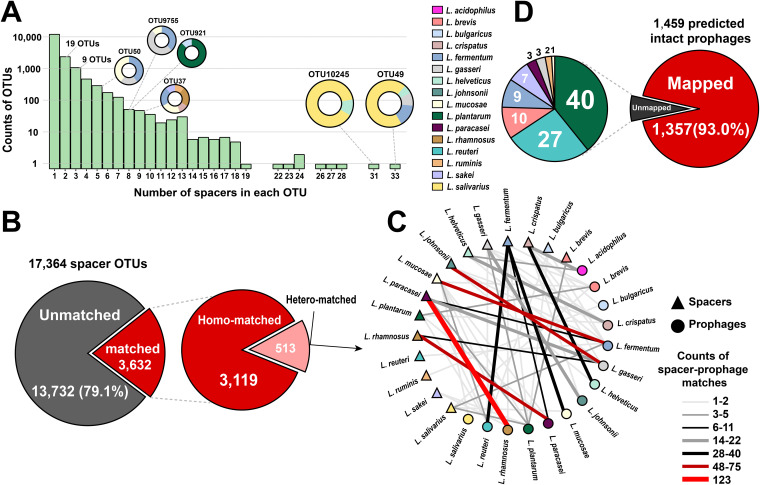
Association between CRISPR spacers and *Lactobacillus* prophages. (A) OTU clustering of CRISPR spacers. Circles on columns represent the OTUs clustered by spacers from multiple species. (B) The proportion of matched spacer OTUs and unmatched spacer OTUs. Matched spacer OTUs were further divided into homo-matched OTUs (the spacer matched the prophage with the same species) and hetero-matched OTUs (the spacer matched the prophage from different species). (C) The spacer-prophage matching network of hetero-matched OTUs. (D) The proportion of mapped prophages and unmapped prophages. Unmapped prophages were further subdivided according to their host species. Source data are provided in Data Set S1, Tab5 and 6.

CRISPR spacers represent the memory of past exogenous gene aggressions, bestowing adaptive immunity on the host (30). Research into the fundamental link between spacers and phages will advance our understanding of phage population diversity and bacteria-phage interactions. Based on the prediction results of CRISPR-Cas systems in lactobacilli, a total of 29,007 spacer sequences were obtained from all CRISPR loci. We performed operational taxonomic unit (OTU) clustering on all spacers; in total, 17,364 OTUs were assigned; however, 12,460 (71.8%) of them contained only one spacer sequence (Fig. 7A). Of the remaining 4,904 OTUs, there were only 34 OTUs containing CRISPR spacers from different species, illustrating that *Lactobacillus* phages are species-specific.

Subsequently, to investigate the origins of these spacers, all extracted sequences were mapped to sequences of the 1,459 predicted intact prophages involved in this study and to those of 81 *Lactobacillus* phages from the NCBI database via nucleotide BLAST searches. Most (79.1%, 13,732/17,364) of the spacer OTUs did not match any (pro)phage (Fig. 7B), suggesting that there is a huge discrepancy between the demonstrable diversity of *Lactobacillus* (pro)phages and the number of known sequences in public databases. Among the 3,632 matched spacer OTUs, 3,119 were paired to prophages within the same species, while 513 CRISPR spacers were paired to prophages from different species. The number of species matching between spacers and prophages is shown in Fig. 7C, and the three pairs with the highest frequency were L. rhamnosus-*L. (para)casei* (*n *= 198), L. gasseri-L. johnsonii (*n *= 84), and *L. fermentum*-*L. mucosae* (*n *= 79), consistent with the results of ANI analysis. This species matching analysis also revealed a global pattern of *Lactobacillus* prophage specificity to their bacterial hosts.

From the perspective of these prophages, 93% (1,357/1,459) of the predicted intact prophages matched one or more spacer OTUs (Fig. 7D), illustrating that the prediction of intact prophages in this study is relatively reliable and accurate. Among the unmapped prophages, the proportion of *L. plantarum* prophages was the highest. The same was observed in the publicly available *Lactobacillus* phages—13 *L. plantarum* phages did not map any spacer (Fig. S4A). It appears that none of the CRISPR-Cas systems in *Lactobacillus* revealed a history of these *L. plantarum* phages infecting bacterial hosts. In addition, 52 (3.6%) intact prophages mapped CRISPR spacers from the same host bacterium (Fig. S4B); although found at a low frequency, self-targeting spacers located within intact prophage regions can be observed in 13 of the 16 species.

10.1128/mSystems.01211-20.4 FIG S4 (A) The proportion of mapped public *Lactobacillus* phages and unmapped phages. (B) The proportion of predicted intact prophages with self-targeting CRISPR spacers. Download FIG S4, TIF file, 2.7 MB. Copyright © 2021 Pei et al. 2021 Pei et al. https://creativecommons.org/licenses/by/4.0/ This content is distributed under the terms of the Creative Commons Attribution 4.0 International license.

## DISCUSSION

We sampled and sequenced a large collection of *Lactobacillus* genomes for prophage analysis and revealed a broad prophage genomic diversity among *Lactobacillus* species. To date, most studies aiming to identify prophage diversity in *Lactobacillus* species have focused on a limited number of strains of a few species (31–33). There was a dire need to carry out a comprehensive genome-wide analysis of prophages in this genus to predict the role of different prophages in their evolution, phenotypic characteristics, and ecology. Based on genomic analysis, a previous study showed the presence of prophages in more than 92% of the genomes of 213 lactobacilli (26). We used seven-times higher the number of genome sequences to provide a more detailed and comprehensive prophage distribution of *Lactobacillus* and illustrated that the distribution of prophages in a bacterial genome is species-specific.

Different species of *Lactobacillus* occupy different habitats, and this may be a possible explanation behind the variation in prophage occurrence in their genomes. Habitat generalists can adapt to diverse habitats, whereas specialists are only adapted to specific habitats depending on their nutritional requirements (34). For instance, in dairy, *Lactobacillus* strains usually rely on a limited amount of sugar for their nutritional needs (35). In the human gut, *Lactobacillus* strains can resist stomach acid and bile salts by activating stress-responsive genes (36). Thus, to occupy varied ecological habitats, habitat generalists conserve as many genes as possible, and also, due to a diverse virome of the human gut, they acquire many new functional genes via (pro)phage-mediated HGT (37). Adaptability to harsh environmental conditions confers flexibility to these species against various stress triggers, which may be a reason behind the presence of many intact prophages in their genomes, further explaining why habitat specificity has a role in the intactness of prophages (38). On the other hand, species that tended to occupy a restricted habitat have relatively condensed genomes (for example, *L. bulgaricus*, ∼1.8 Mb; L. crispatus, ∼2.0 Mb. L. helveticus, ∼2.0 Mb; *L. ruminis*. ∼2.0 Mb) because they do not need to respond to many environmental changes (39, 40). It has been demonstrated that gene loss and genome reduction are the events that happen during the evolution of habitat specialists (41); hence, we observed fewer intact prophages but more incomplete prophage fragments in restricted habitat-derived species. In addition, regarding the uneven distribution of intact prophages in *Lactobacillus* strains isolated from different sources, we similarly assume that it may be related to the environmental pressure on strains. The human or mammalian intestine is considered a relatively stable environment in which intestinal peptides, mucosal barriers, and other substances together maintain homeostasis, whereas the living environment of strains isolated from fermented foods is unstable and sometimes harsh. For example, phage populations in food change rapidly and get reshaped approximately every few days (42), and strains may get exposed to a variety of microorganisms from air, water, and soil, which contain various kinds of phages, making them more susceptible to phage infection. Canchaya et al. (43) found that Lactococcus lactis strains used for cheese fermentation are under extreme pressure from phages, thus carrying more prophages. Therefore, it appears that lactobacilli tend to integrate more prophages in response to a variety of environmental stresses.

*Lactobacillus* prophages display wide variations in their genome sizes and GC contents. Several factors influence the genome size of prophages; for example, some bacterial genes are incorporated into prophage genomes during the replication process (44), causing an increase in their genome size. Conversely, the host may selectively discard certain redundant genes, which may shorten the prophage fragment length. The significant differences in GC content between *Lactobacillus* prophages and their hosts also attracted our attention. However, we cannot confirm whether the GC content of *Lactobacillus* prophage tends to change and whether the difference in GC content is related to the host species; the key driver of this phenomenon remains enigmatic and needs further investigation. ANI analysis showed that *Lactobacillus* prophages present a discontinuity of genetic diversity, for mycobacteriophages, which are relatively broad-spectrum, showing a continuum of genetic diversity and an open population system (45), whereas for *Lactobacillus* prophages, the limited similarities between clusters indicate that phage infection may be highly species-specific in the genus *Lactobacillus*, while the greatly varied diversity of individuals in each cluster likely reflects the dominance of strain-specific infections. Moreover, the integration site and integrase can also be considered indicators to discuss the genomic diversity and host specificity of prophages (46). Brueggemann et al. reported that pneumococcal complete prophages were consistently inserted into specific locations within the genome (47) because the prophage transmission across bacterial strains is quite common in Streptococcus pneumoniae. The highly diverse prophage integration sites in *Lactobacillus* genomes also demonstrate the diversification and strain specificity of *Lactobacillus* prophages.

The prevalence of ARGs in *Lactobacillus* is well known, and many strains show phenotypic multidrug resistance (48). Foodborne drug-resistant bacteria may promote the transfer of ARGs to other commensal microorganisms in the human gastrointestinal tract (49), thus posing a threat to public health and food safety (50), while a complete prophage is a crucial carrier and reservoir of ARGs (51), leading to the widespread dissemination of antibiotic resistance (52). Therefore, ubiquitous prophages should arouse our attention to assess the risk of antibiotic resistance transfer in *Lactobacillus*. We found that the numbers and types of ARGs carried by prophages harbored by different host species were highly variable, particularly the human-derived *Lactobacillus* species; this is likely a reflection of antibiotic overexposure in the clinic. Moreover, we discovered that the potential ARGs in *L. plantarum* were possibly conferred on hosts by prophages and likely contribute to the resistance phenotypes. Although some scholars demonstrated that the majority of intestinal-derived bacterial ARGs are rarely shared with pathogens (53) and the frequency of bacteriophages carrying ARGs is overestimated (54), evaluation of the transfer risk of these ARGs carried by prophages in lactobacilli need further experimental confirmation. Based on the findings in this study, we suggest that when lactobacilli are used as oral dietary supplements and clinical treatments, individuals harboring prophages with ARGs should be treated with caution, and an in-depth evaluation of the safety issues of antibiotic resistance must be conducted to provide more evidence for the safety of using live microorganisms.

The CRISPR-Cas system is a simple and powerful tool in genetic engineering research. Several large cohort studies on the mining and characterization of CRISPR-Cas systems were performed to provide plentiful resources for exploring phage-host associations (55). Crawley et al. (56) have reported that *Lactobacillus* genomes showed a myriad of CRISPR-Cas systems, but the occurrence rates are varied in different species. However, their findings seem to somewhat contradict ours. In this study, the CRISPR-Cas occurrence rates in *L.* (*para*)*gasseri*, *L.* (*para*)*casei*, *L. plantarum*, L. rhamnosus, and *L. salivarius* were much higher than those described by Crawley et al., while L. crispatus, *L. fermentum*, and *L. mucosae* carried CRISPR-Cas systems much less frequently. We expanded the available genomes and presented a more detailed distribution of CRISPR-Cas systems in these *Lactobacillus* species; thus, the results reported here may be more accurate. Additionally, we discovered that self-targeting CRISPR spacers are not rare in *Lactobacillus* prophages. The same phenomenon was observed in Lactobacillus buchneri strains. Nethery et al. (57) suggested that the presence of self-targeting spacers indicates phage escape from immune attack by the CRISPR-Cas system during infection. Alternatively, Nobrega et al. (58) proposed another interpretation that CRISPR-Cas systems accidentally acquire these spacers after these prophages have already been integrated into the bacterial hosts. In either case, our results reflect the inextricable links between prophages and CRISPR systems.

We carried out a comprehensive identification of CRISPR-Cas systems by analyzing 1,472 *Lactobacillus* genomes in an attempt to investigate the relationship between the occurrence of CRISPR-Cas systems and *Lactobacillus* prophages. Our analyses show that the presence of type II systems does not appear to effectively inhibit the integration of intact prophages. On the contrary, lactobacilli carrying type I or type III systems harbored fewer intact prophages in their genomes. At present, there is no sufficient and reliable evidence to confirm this intriguing finding; additional studies are warranted to determine why different types of CRISPR-Cas systems in *Lactobacillus* have different interference efficiencies against temperate phage integration. Here, we put forward some hypotheses. During the lengthy procedure of coevolution between bacteriophages and bacteria, several prophages acquire anti-CRISPR genes as a countermeasure to inhibit CRISPR-Cas systems (59, 60). Rollie et al. (61) also proposed that CRISPR-Cas immune systems may not eliminate the temperate phages when challenged with them, and this imperfect targeting seems to frequently occur in prokaryotes. We speculated that some *Lactobacillus* prophages could carry genes encoding type IIA CRISPR-Cas9 inhibitor proteins, leading to a reduction in the efficiency of type IIA systems for targeted prophage excision. Notably, the resistance of bacteria to bacteriophages is determined not only by one defense system, namely, the CRISPR-Cas system, but also by restriction-modification systems (62), abortive infection systems (63), and other novel systems whose action mechanisms are yet to be revealed (64–66). They are also important components of the bacterial defense system against prophages. We provide insights into the potential association between *Lactobacillus* prophages and CRISPR-Cas systems based on bioinformatics analysis. It is expected that the specific mechanisms of defense against prophages in *Lactobacillus* will be further explored and adapted using useful biotechnological tools in the future.

Of course, the shortcomings of this study should also be stated. First, our entire research of *Lactobacillus* prophages was based on the prediction by PHASTER, and the kernel of the prediction algorithm of PHASTER is based on the sequence similarity alignment. The constructed phage protein database was used to annotate the bacterial genome, presenting the phage gene regions with clustering characteristics, and considering the completeness of each predicted prophage region. That is, if the cornerstone genes or phage-like genes of a certain prophage are not included in the existing database, it may be considered an incomplete prophage or even fail to be detected. Similarly, as most CRISPR spacers do not produce significant alignments to any known phage genome, the insufficiency of viral sequence databases is a common problem faced by researchers. Thus, the number of predicted intact prophages in lactobacilli might be underreported; the exact range of the actual complete prophage also could not be accurately defined. To promote the field of bacteriophage to cross the technological barrier and then develop rapidly, we expect more and more active phage genomes to be mined and novel tools in predicting active prophages with higher accuracy to be developed. Second, the prediction of prophage is possibly affected by the quality and integrity of the input genomes; draft genomes with a large number of fragments could reduce the accuracy of prediction. But the progress in prediction tools and assembling technology will bring us more interesting discoveries of the prophage. Third, out of our interest in whether these predicted intact prophages in this study can be induced and actually transformed on the bench, in another study (11), we selectively induced 142 strains (involved in this study) from 6 species and successfully obtained several inducible temperate phages. Almost all of the induced phages matched the corresponding intact prophage regions predicted by PHASTER. However, due to the different sensitivity of lactobacilli to MMC concentration (10), we cannot determine whether that unresponsive predicted intact prophage is induction failure, inactivated, or false positive. Therefore, although we provide a reduced data set of predicted intact prophages, the activity of them should be carefully evaluated when considering whether *Lactobacillus* lysogens might be used in any fermentation industries or probiotic productions.

In conclusion, this study presented a comprehensive screening of prophages in 1,472 *Lactobacillus* genomes belonging to 16 different *Lactobacillus* species and showed the presence of a wide variety of prophages. We observed an uneven prophage distribution, with highly diverse genome features and distinct clusters based on host species, with a better understanding of *Lactobacillus* prophage genetic diversity. Moreover, the detection of ARGs in *Lactobacillus* prophages provided valuable data and the basis to determine the safety and development of *Lactobacillus* for agricultural and human nutritional applications. Notably, type I and III CRISPR-Cas systems are possibly one of the effective strategies in counteracting prophages for lactobacilli. This study’s results could be of interest to all biotechnological and clinical fields that require a better safety assessment and functional characterization of *Lactobacillus.*

## MATERIALS AND METHODS

### *Lactobacillus* genome collection.

Of the 1,472 genomes of *Lactobacillus* included in this study, 1,001 strains were originally isolated and sequenced in our laboratory. The remaining 471 assembled genomes were acquired from the National Center for Biotechnology Information (NCBI) database (https://www.ncbi.nlm.nih.gov/genome/?term=Lactobacillus). These *Lactobacillus* genomes represented 16 different species (>30 genomes were available for each species)—L. acidophilus (*n *= 35), L. brevis (*n *= 69), *L. bulgaricus* (*n *= 38), L. crispatus (*n *= 97), *L. fermentum* (*n *= 140), *L.* (*para*)*gasseri* (*n *= 116), L. helveticus (*n *= 54), L. johnsonii (*n *= 53), *L. mucosae* (*n *= 122), *L. plantarum* (*n *= 134), *L.* (*para*)*casei* (*n *= 147), L. rhamnosus (*n *= 96), L. reuteri (*n *= 88), *L. ruminis* (*n *= 95), *L. sakei* (*n *= 44), and *L. salivarius* (*n *= 144). The specific information of the strains is listed in Table S1A.

### DNA extraction, genome sequencing, and draft assembly.

All *Lactobacillus* strains were cultured in liquid DeMan-Rogosa-Sharpe (MRS) medium (Sinopharm Chemical Reagent Ltd., Shanghai, China) and incubated at 37°C for 12 to 24 h. The bacterial culture was centrifuged at 6,000 rpm for 3 min, and then the supernatant was collected. Next, bacterial cells were washed in 0.9% sterile normal saline and collected by centrifugation under the same conditions. Genomic DNA extraction was performed using the rapid bacterial genomic DNA isolation kit (Sangon Biotech Ltd., Shanghai, China) according to the instruction manual.

Genome sequencing was performed using the Illumina HiSeq× 10 platform (Novogene Biotech Ltd., Tianjin, China; Majorbio Biotech Ltd., Shanghai, China), which generated 2 × 150-bp pair-end read libraries. For each sample, the raw data with no less than 100× genome coverage depth were provided and trimmed into high-quality reads (clean data). The software SOAPdenovo2 (67) was used to assemble contigs, and then we tested various kmer values and obtained the optimal assembly result. Next, according to the relationship between paired-end reads and read overlaps, the assembly result was partially assembled and optimized to form scaffolds. The quality data of each genome (genome size, GC content, genome level, number of scaffolds, and *N*_50_ value) are listed in Table S1A.

### Prophage prediction.

PHAge Search Tool enhanced release (PHASTER; http://phaster.ca/) (68, 69) was used as a predictor to identify prophages within the genomes of 1,472 *Lactobacillus* strains and provide the location, region length, GC content, and phage-related gene annotation of each prophage. As described in the software, for each predicted prophage region, the scoring criterion was as follows: if a predicted prophage region completely covered a certain phage organism in the database, it was marked with a total score of 150. If not, the following two methods were used. (i) If more than half of the coding sequences (CDS) in this region matched the CDS of a certain phage organism, this proportion was calculated and then multiplied by 100, and the query coverage of the region with that target phage was calculated and then multiplied by 50. The total score of the predicted prophage region is the sum of the two items. (ii) If any of the specific phage-related genes (such as integrase, transposase, protease, terminase, portal, capsid, head, tail, fiber, coat, plate, or lysin) was present, the score was increased by 10 for each gene detected. If this predicted prophage region met the criteria genome size > 30 Kb, CDS number > 40, and proportion of phage-related proteins and hypothetical proteins > 70%, the score was increased by 10 for each fulfilled criterion. By comparing the total scores of the two methods, the higher score was chosen as the final score of the region. A region with a score of <70 was marked as an incomplete prophage; a region with a score ranging from 70 to 90 was marked as a questionable prophage, and a region with a score of >90 was marked as an intact prophage.

### Calculation of ANI and heat map visualization.

ANI values were calculated using the program JSpecies v1.2.1 (70). When a prophage genome was compared to itself, the ANI value was marked as 100%. When orthologous gene similarity between two prophages was found to be less than 60.0%, it was uniformly recorded as NA. Heat map visualization and hierarchical clustering were performed using HemI (Heatmap Illustrator v1.0) (71).

### ARG prediction and antibiotic susceptibility testing.

Open reading frames of *Lactobacillus* prophage genomes were predicted using GeneMarkS (http://topaz.gatech.edu/GeneMark/) and then were assigned to the Comprehensive Antibiotic Resistance Database (CARD; http://arpcard.mcmaster.ca) (72) using a blastp alignment (BLAST v2.2.28+). A conservative threshold (amino acid identity, ≥30%; comparison coverage, ≥70%) was used to identify putative ARGs (73).

The MIC values of nine antibiotics (erythromycin, clindamycin, ciprofloxacin, rifampin, amoxicillin, neomycin, gentamicin, kanamycin, and streptomycin; purchased from Sangon Biotech Ltd., Shanghai, China) against *Lactobacillus* species were measured using the broth microdilution method according to ISO 10932:2010 with slight modifications (74). In brief, *Lactobacillus* strains (*L. plantarum*, *n *= 115; *L. paracasei*, *n *= 121; and L. rhamnosus, *n *= 71) were grown in MRS liquid medium at 37°C for 12 to 24 h. Before susceptibility testing, all strains were propagated for two generations. Hand-made 96-well plates were used for serial 2-fold dilutions of the nine antibiotics. The dilutions of 100 μl were distributed into each well. The bacterial suspensions were diluted until the optical density (OD) was between 0.16 and 0.2 at 625 nm, with a corresponding concentration of 3 × 10^8^ CFU/ml. The suspensions were diluted again 10 times, and 100 μl was added into each well. The 96-well plates were placed in an anaerobic atmosphere and incubated for 48 h, after which the OD was measured at 625 nm with a Multiscan Spectrum device (Thermo Fisher Scientific). The endpoint values were defined as the minimum antibiotic concentration with no detectable growth. The interpretation criteria used to differentiate susceptible strains from resistant strains were determined by referring to the microbiological breakpoints recommended by the European Food Safety Authority (EFSA) (75).

### CRISPR-Cas system identification and spacer analysis.

The CRISPRCasFinder program (https://crisprcas.i2bc.paris-saclay.fr/CrisprCasFinder/Index) (76) was used to search for CRISPR arrays and repeat sequences. MacSyFinder (77) was used to identify Cas genes and the CRISPR-Cas type or subtype. A spacer OTU is defined as a group of the same spacer. OTU clustering and spacer-prophage matching were both performed using local nucleotide BLAST searches.

### Data statistical analysis and visualization.

The Kruskal-Wallis test and the Mann-Whitney U test were performed using SPSS PASW Statistics v18.0. The *t* test (*n* < 30) and *z* test (*n* ≥ 30) were analyzed using Microsoft Office Excel 2016. The Tukey’s honestly significant difference (HSD) test was performed using GraphPad Prism v8.0.

The stacked bar charts, pie charts, box-whisker plots, and violin plots were visualized using GraphPad Prism v8.0. The line graphs were plotted using Microsoft Office Excel 2016. Genomic organization of predicted intact prophages was visualized using IBS v1.0.3 (78). The networks were visualized with Cytoscape software (79). The UpSetR package v1.4.0 (80) was used to visualize the upset plot. All figures were further edited using Adobe Illustrator CC2020.

### Data availability.

Of the 1,001 *Lactobacillus* strains deposited in the NCBI GenBank database, 391 were released as part of our previous studies (22, 81–84), and the remaining 610 genomes were deposited under project accession no. PRJNA658852. The accession numbers for all individual genomes used in this study (including 471 genomes downloaded from NCBI) are presented in Table S1A. The accession numbers for 81 *Lactobacillus* phage genomes used in this study are presented in Table S1B.

## Supplementary Material

Reviewer comments
