# Supplementary figures and images for: Comprehensive Scanning of Prophages in Lactobacillus: Distribution, Diversity, Antibiotic Resistance Genes, and Linkages with CRISPR-Cas Systems
Source: mSystems. 2021 Jun 1;6(3):e01211-20. doi: 10.1128/mSystems.01211-20 (PMC8269257; doi:10.1128/mSystems.01211-20)

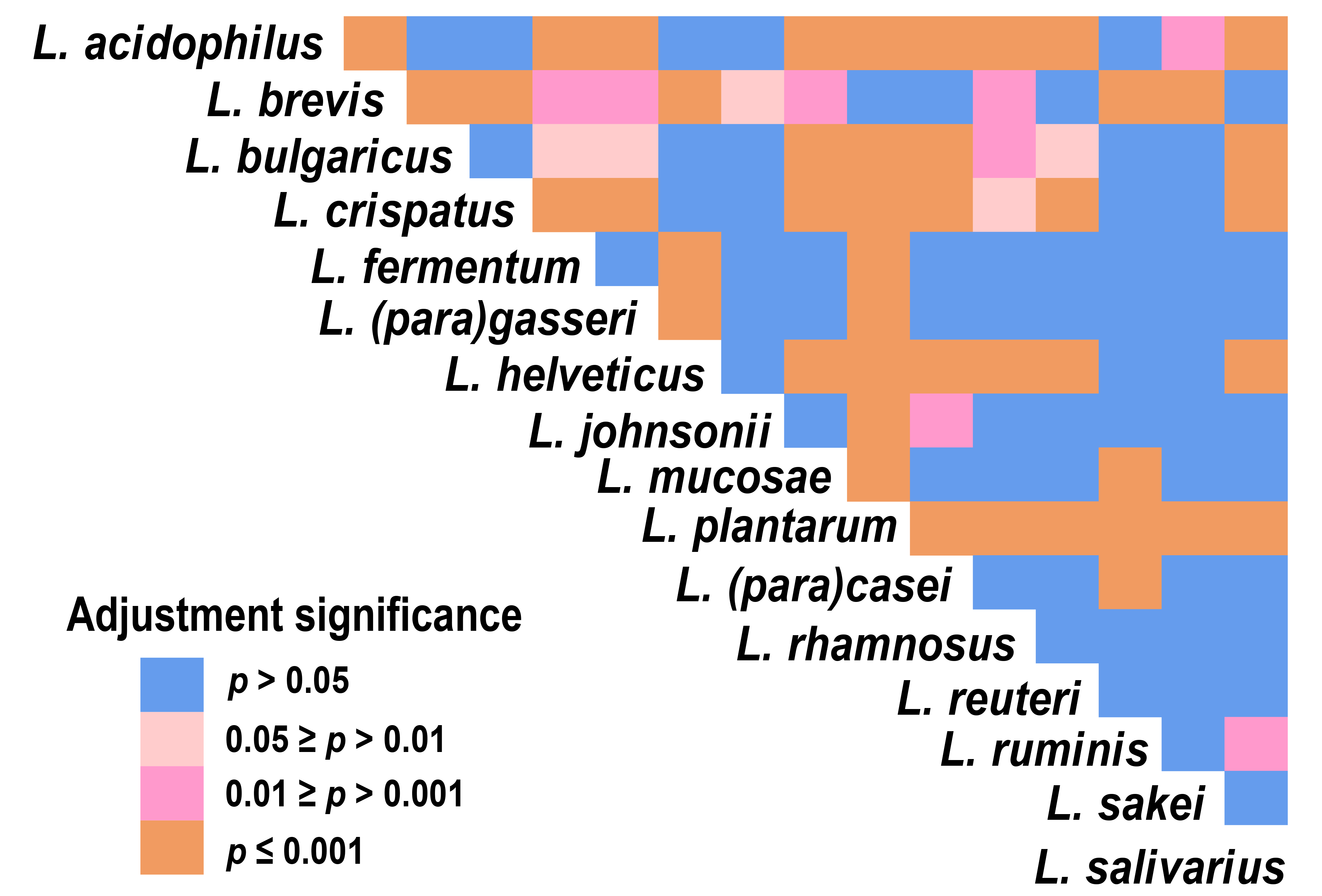

Supplement: FIG S1 [file msystems.01211-20-sf001.tif]

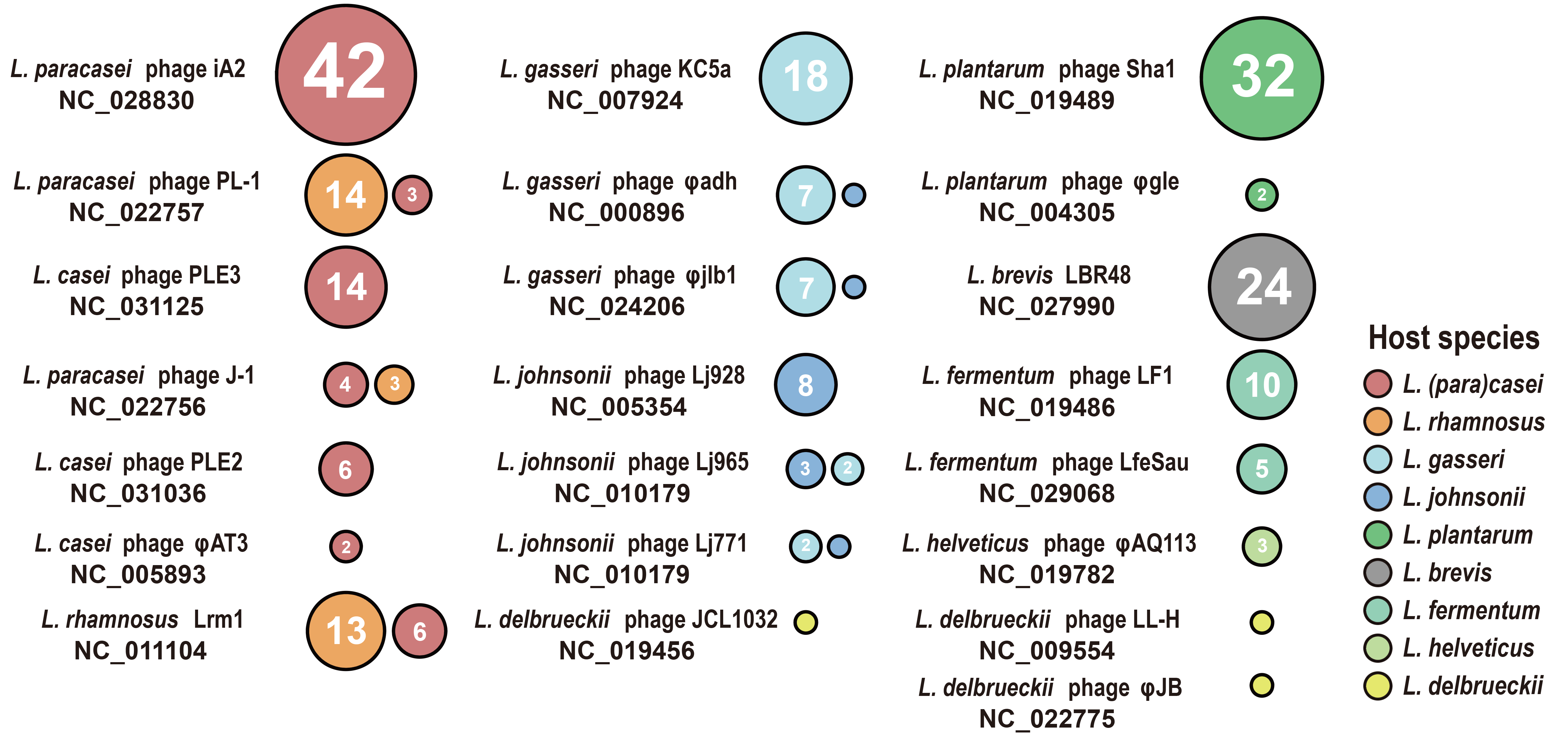

Supplement: FIG S2 [file msystems.01211-20-sf002.tif]

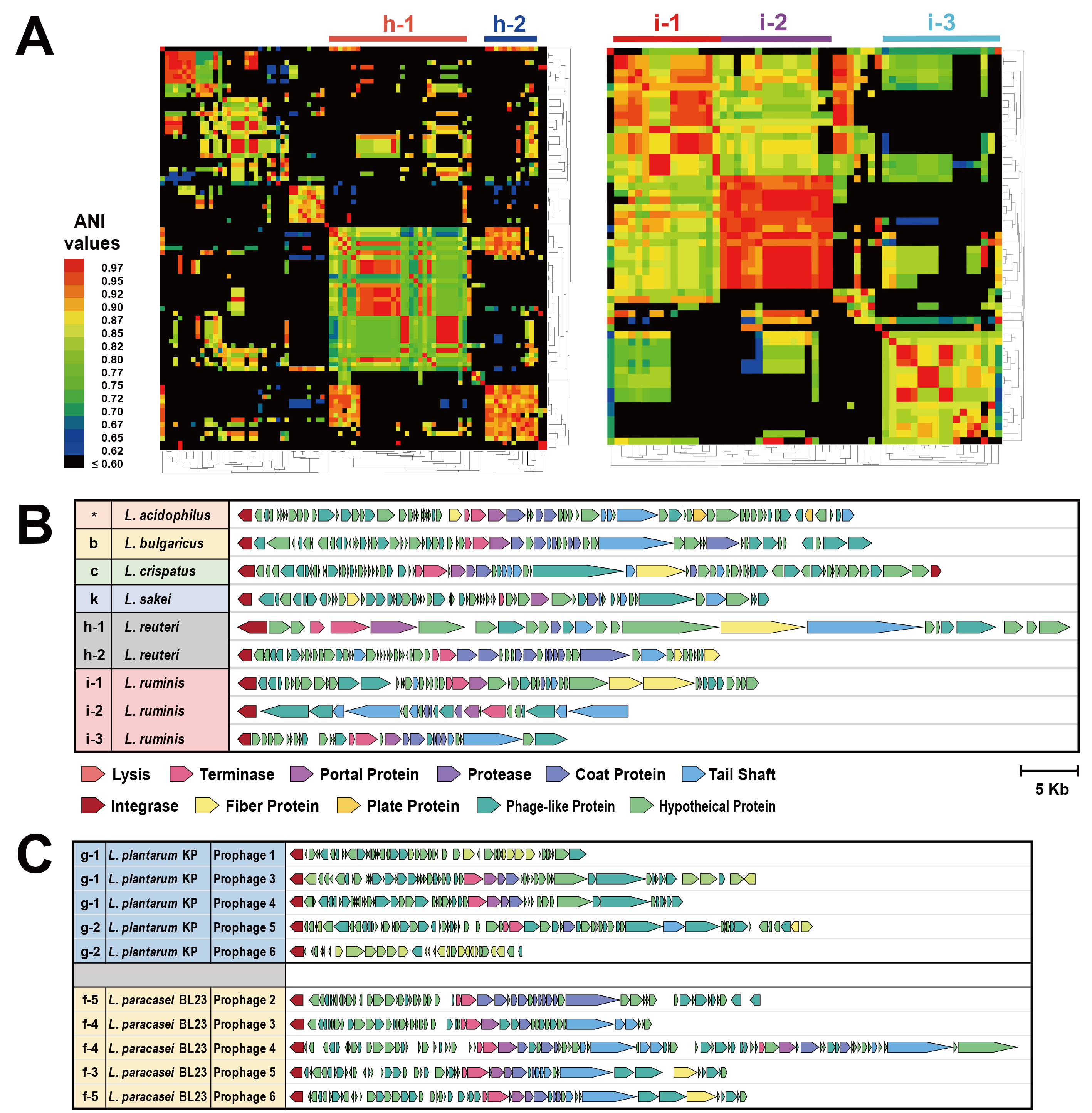

Supplement: FIG S3 [file msystems.01211-20-sf003.tif]

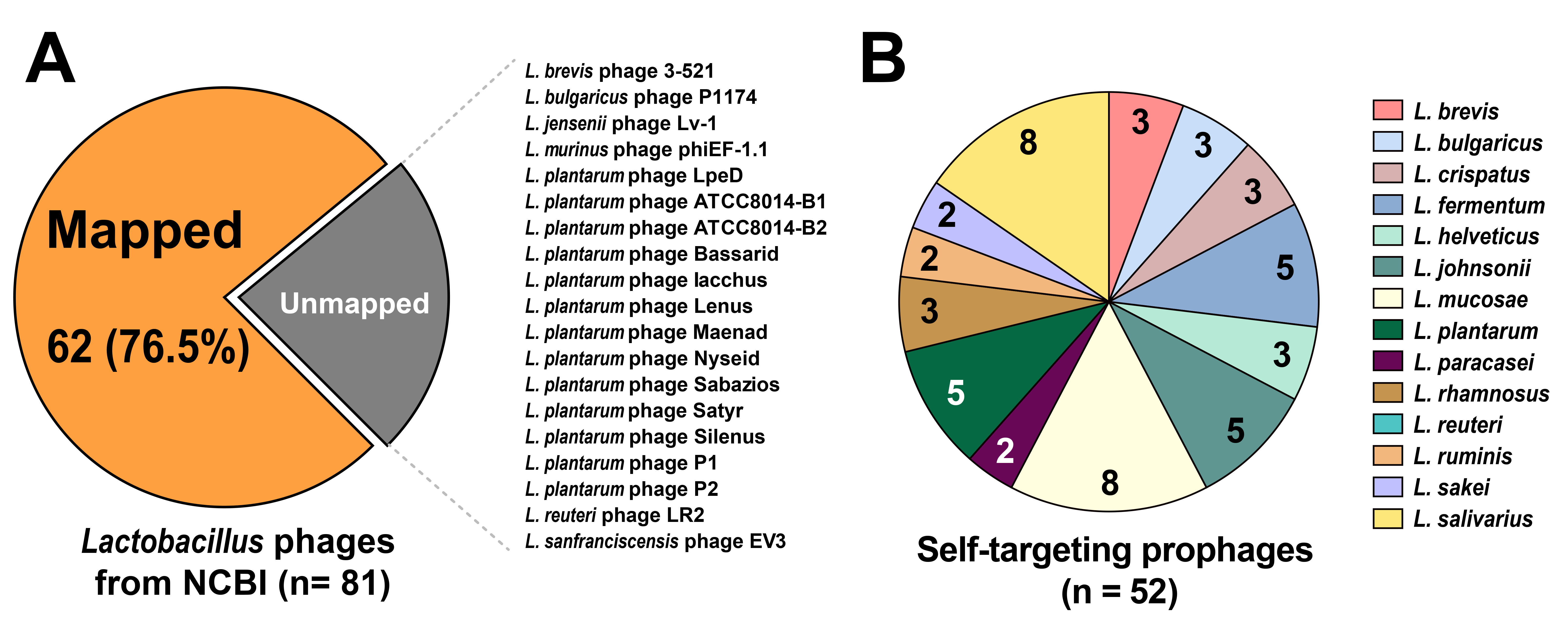

Supplement: FIG S4 [file msystems.01211-20-sf004.tif]
